# Activity and mechanism of naringin in the treatment of post-infectious cough

**DOI:** 10.1186/s12890-025-03788-6

**Published:** 2025-07-09

**Authors:** Yinghan Chen, Dong Wang, Meng Song, Weiqi Pan, Wenda Guan, Zifeng Yang, Shan Zhong

**Affiliations:** 1 State Key Laboratory of Respiratory Disease, Guangzhou Medical University, National Clinical Research Center for Respiratory Disease, Guangzhou Medical University, Guangzhou Institute of Respiratory Health, Guangzhou Medical University, The First Affiliated Hospital, Guangzhou Medical University, Guangzhou, 510080 China; 2 School of Traditional Chinese Materia Medica, Shenyang Pharmaceutical University, Shenyang, 110016 China; 3 Guangzhou National Laboratory, Guangzhou, 510005 China

**Keywords:** Naringin, Post-infectious cough, Network pharmacology, Anti-inflammatory, Antioxidant

## Abstract

**Objective:**

To explore activity and mechanism of naringin in the treatment of PIC (Post-Infectious Cough) by virtue of network pharmacology and animal studies.

**Methods:**

The targets associated with naringin were obtained from the SwissTargetPrediction and Super-PRED databases. Disease-related targets were collected from GeneCards and OMIM (Online Mendelian Inheritance in Man). Venny was utilized to identify the overlapping targets between naringin and the disease. PPI (Protein–Protein Interaction) networks for disease-related targets were constructed using STRING and Cytoscape 3.10.1. GO (Gene Ontology) functional annotation and KEGG (Kyoto Encyclopedia of Genes and Genomes) pathway enrichment analyses were performed with Metascape. Molecular docking between key targets and naringin was conducted using AutoDock Vina. In the animal experiments, PIC models were established in guinea pigs via intranasal inoculation with A/PR/8 virus, and cough frequency was measured after citric acid-induced coughing. Morphological changes in lung tissue and airways were assessed using the HE (Hematoxylin–Eosin) staining method. The relative expression levels of IL-8 (Interleukin-8), IL-1β (Interleukin-1β), TNF-α (Tumor Necrosis Factor-Alpha), and NF-κB p65 (Nuclear Factor Kappa-B p65 Subunit) mRNA were analyzed by RT-qPCR (Reverse Transcription Quantitative Polymerase Chain Reaction). SOD (Superoxide Dismutase) activity in lung tissue was measured using a colorimetric assay.

**Results:**

After screening, naringin may contribute to the treatment of post-infectious cough by targeting proteins expressed by core genes such as HSP90AA1 (HSP 90-Alpha), TLR4 (Toll-like Receptor 4), MTOR (Mechanistic Target of Rapamycin), HIF1A (Hypoxia-Inducible Factor Alpha), and NF-κB1 (Nuclear Factor Kappa-B Subunit 1). KEGG enrichment analysis revealed involvement in pathways including the HIF-1 (Hypoxia-Inducible Factor 1) signaling pathway and the PD-L1 (Programmed Death-Ligand 1) expression and PD-1 (Programmed Cell Death Protein 1) checkpoint pathway in cancer. Molecular docking results indicated that naringin exhibited strong binding affinity with HSP90AA1, TLR4, MTOR, HIF1A, NF-κB1, NOS3 (Nitric Oxide Synthase 3), and GRB2 (Growth Factor Receptor-Bound Protein 2). In animal experiments, compared to the normal group, guinea pigs in the model group exhibited a significantly higher number of coughs, pronounced lung tissue hyperplasia, and inflammatory cell infiltration. Additionally, the relative expression of IL-8, IL-1β, TNF-α, and NF-κB p65 mRNA was significantly increased, while lung tissue SOD activity was decreased. Treatment with naringin significantly reduced the number of coughs, attenuated pathological changes in lung tissue, lowered the lung index, decreased the relative expression of IL-8, IL-1β, TNF-α, and NF-κB p65 mRNA, and significantly increased SOD activity in lung tissue compared to the model group.

**Conclusion:**

Naringin shows therapeutic potential to alleviate PIC symptoms in a guinea pig model through anti-inflammatory and antioxidant mechanisms.

## Background

After the resolution of acute-phase symptoms of a respiratory viral infection, patients may continue to experience a prolonged cough, clinically referred to as PIC [1]. PIC typically presents as an irritating dry cough or a cough producing scant white mucus sputum, lasting between 3 to 8 weeks (subacute cough), with some cases persisting beyond 8 weeks, resulting in chronic cough that significantly impairs the patient's quality of life. However, the Chinese national guideline on diagnosis and management of cough (2021) does not recommend specific medications specifically for PIC [2]. The etiology of PIC remains incompletely understood in both Chinese and Western medicine. Western medicine posits that PIC is generally self-limiting and may not require treatment in mild cases; in severe cases, antihistamines, cough suppressants, or corticosteroids may be employed, though these medications often have numerous side effects, limited overall efficacy, and may lead to refractory cough [3]. TCM (Traditional Chinese Medicine) offers unique advantages in symptom improvement and disease course reduction, emphasizing a holistic and evidence-based approach. Nonetheless, existing clinical studies are superficial, lacking in-depth exploration and analysis of therapeutic mechanisms and pharmacological effects [4].

Naringin, a natural flavonoid glycoside predominantly found in citrus fruits of the Brassicaceae family and in Chinese herbal medicines such as Hovenia dulcis, Fructus Forskohlii, and Pericarpium Citri Reticulatae, exhibits various pharmacological activities including anti- inflammatory, antioxidant, and anticancer effects, garnering significant attention in both TCM and modern pharmaceutical research [5]. Furthermore, evidence has demonstrated that naringin exerts antitussive effects in physiologically induced experimental cough, chronic airway inflammation, and cough triggered by airway neurogenic inflammation. Although extensive studies have been conducted on naringin, its intervention mechanisms in PIC remain unreported. To address this gap, the present study established a PIC model and employed network pharmacology prediction combined with animal pharmacological validation to investigate the inhibitory effects of naringin on PIC.

## Materials and methods

### Network pharmacology

#### Target collection of naringin

The SMILES structure of naringin was retrieved from the PubChem database [6] (https://pubchem.ncbi.nlm.nih.gov/) and used to predict its target information via the SwissTargetPrediction database [7] (http://swiss-targetprediction.ch/) and the SuperPred database [8] (https://prediction.charite.de). Targets with probability values greater than 0.1 were selected as constituent targets based on the prediction thresholds.

#### Disease target search and acquisition

Target screening for post-infectious cough was conducted using the keyword “post-infectious cough” to predict target genes from the GeneCards [9] (https://www.genecards.org/.) and OMIM [10] (http://www.omim.org/) databases. The identified target genes from these databases were consolidated, duplicates were removed, and the list was cross-referenced and validated using the UniProt Protein Database [11] (https://www.uniprot.org/.), resulting in the final set of target genes associated with post-infectious cough. The intersection targets between naringin and disease-related genes were identified using Venny 2.1 (https://bioinfogp.cnb.csic.es/tools/venny/), and a Venn diagram was generated to visualize the overlapping targets.

#### Network visualization construction and analysis

The component-disease intersection targets, along with the corresponding components and diseases, were imported into Cytoscape 3.10.1 for mapping analysis to construct a disease-component-target network diagram, visualizing the relationships among these elements Table 1.

**Table 1:** Primer sequence for RT-qPCR

Target Gene	Direction	Sequence (5′–3′)
IL-8	Forward primer	GAGGGTATGGTCGTGACAAAGTTG
	Reverse primer	GGTGGAAAGGTGTGGTGTGAATC
IL-1β	Forward primer	GCTGGAGAGTGTAGATGGCAAAC
	Reverse primer	GTTCTGCTTGAGAGGTGCTGAT
TNF-α	Forward primer	AACCAGCAAGCAGAGGAGGAG
	Reverse primer	GAGGTACAGCCCATCCGAAGG
NF-κB p65	Forward primer	GGAATGCGGTTCAGATACAAATGTG
	Reverse primer	GCGATTGTCCTCTGTGCTGTG
GAPDH	Forward primer	TGGTGAAGCAGGCATCAGAGG
	Reverse primer	TGGAAGAATGGCTGTCACTGTTG

#### PPI network construction

The component-disease intersection targets were uploaded to the STRING database [12] (https://cn.string-db.org/) to generate the PPI network for naringin and post-infectious cough shared targets. The PPI network analysis was performed with the confidence level of the STRING database set to 0.4 (medium confidence) and disconnected nodes hidden. The resulting PPI network was further visualized and analyzed using Cytoscape 3.10.1, where the CytoNCA plug-in was applied to evaluate the network's topological properties and identify key core targets. Core targets were selected based on three topological parameters—Closeness, Betweenness and Degree—with a threshold set to values above the median for each metric. These filtered targets were subsequently defined as key therapeutic targets for naringin in treating post-infectious cough.

#### GO gene enrichment analysis and KEGG pathway analysis

Go and KEGG pathway enrichment analyses were performed using Metascape [13] (http://metascape.org). Shared targets between naringin and post-infectious cough were analyzed with default parameters (Minimum overlap = 3, *P*-value cutoff = 0.01, Minimum enrichment factor = 1.5) and species set to “Homo sapiens”. Enriched terms were ranked by ascending *P*-value, with the top 10 pathways (or all pathways if fewer than 10) selected for visualization. The visualization results are generated through the Wei Sheng xin platform (https://www.bioinformatics.com.cn/).

#### Molecular docking

Key target proteins were identified through network centrality analysis using three metrics: Degree, Closeness and Betweenness. The average values across all nodes in the PPI network were calculated as Degree = 7.10, Closeness = 22.08, and Betweenness = 46.38. Targets exceeding all three thresholds (Degree ≥ 7.10, Closeness ≥ 22.08, and Betweenness ≥ 46.38) were selected as core candidates, ultimately identifying seven key targets: HSP90AA1, TLR4, MTOR, HIF1A, NF-κB1, NOS3, and GRB2. The PDB (Protein Data Bank) structures of these key target proteins were retrieved from the PDB database and preprocessed using AutoDock Tools to generate PDBQT files by removing water molecules, adding polar hydrogens, and assigning Gasteiger charges. Grid box dimensions and coordinates for each target were customized to cover their active sites, with detailed parameters listed alongside binding energies in Table 2. Binding energies between naringin and these targets were calculated using AutoDock Vina, with lower binding energies indicating higher structural stability. Finally, the interactions were visualized and analyzed using PyMOL software.

### Animal experimental validation

#### Animals and reagents

Specific pathogen-free female Hartley guinea pigs weighing 250–300 g were procured from the Guangdong Provincial Medical Laboratory Animal Center, China. The influenza A/PR8/34 (H1N1) virus strain (PR8) was sourced from the Guangdong Provincial Center for Disease Control and Prevention. TCID_50_ (The viral 50% tissue culture infective dose) was calculated using the Reed–Muench method and determined to be 10⁻⁷/100 μL. Naringin was purchased from Chengdu Ruens Biotech Company. Asm (Asmeton, China Standard of National Drug, H20033669) was provided by Daiichi Sankyo Pharmaceutical Co. Ltd. (Shanghai, China). Citric acid was purchased from Sigma Aldrich (Seattle, USA). RT-qPCR premix and quantitative PCR kits were provided by Novozymes Biotechnology Co. (Nanjing, China). Primers were synthesized by Sangon (Shanghai, China).

#### Grouping, modeling and dosing

SPF-grade female guinea pigs, aged 6 to 12 weeks, were housed in individually ventilated cages at the Zhongshan Development Zone Laboratory, China National Analytical Center in Guangzhou. The animal study received ethical approval from the same institution, adhering to established animal care guidelines and approved protocols Table.

After a three-day acclimatization period, a citric acid cough sensitivity assay was performed to identify and exclude guinea pigs with abnormal cough frequencies from the study. The remaining animals were randomly assigned to six groups: the control group (0.5% carboxymethyl cellulose sodium), A/PR/8 group, A/PR/8 + ASM (21.62 mg/kg) group, and A/PR/8 + Naringin low, medium, and high dosage groups (50, 100, and 200 mg/kg)——doses selected based on validated effective ranges from antitussive and chronic bronchitis studies (intravenous: 15–60 mg/kg; oral: 9.2–36.8 mg/kg) [14, 15], where the medium dose (50 mg/kg) aligned with the upper limit of oral efficacy reported in prior work, and the high dose (200 mg/kg) aimed to explore potential saturation effects in peripheral pathways while ensuring safety through short-term administration (7 days) supported by 8 week oral tolerance data [15]. This randomization ensured comparable baseline cough frequencies across all groups. The PIC model was established using A/PR/8 virus nasal drops. Except for the control group, the drug was administered 2 h after the nasal drop for seven consecutive days.

#### Evaluation of cough sensitivity

In this experiment, guinea pigs were placed in the whole-body plethysmograph and assess cough sensitivity, which was evaluated on the seventh days post-viral infection. Cough induction was achieved using 1 ml of 0.4 M citric acid, nebulized over six minutes. A cough was identified by specific behaviors such as the guinea pig stretching its front legs, extending its neck, and opening its mouth, accompanied by a distinctive sound.

#### Anesthesia and euthanasia

All animals were deeply anesthesia with isoflurane at an induction concentration of 3%−5%, and the adequacy of anesthesia was confirmed by the absence of toe pinch reflex. After complete unconsciousness was achieved, cardiac exsanguination was performed until the complete cessation of vital signs.

#### HE staining

Following seven days of naringin treatment, lung tissues and bronchi were excised. A portion of the tissue was fixed in 4% formaldehyde, embedded in paraffin, and stained with HE for optical microscopic examination of inflammatory cell infiltration around the blood vessels, and lung parenchyma.

#### RT-qPCR

Total RNA was extracted from the lung tissues using Trizol reagent. Then, the total RNA was reversely transcribed to cDNA, and strictly following the instructions of the reagent.

#### Detection of pulmonary oxidative stress indicators

SOD was detected by taking lung tissue homogenate, and the procedure and method were strictly in accordance with the instruction of the kit.

#### Statistical analysis

Statistical analysis was performed using the Student’s two-tailed test for two-group comparisons and one-way ANOVA (Analysis of Variance) for multiple groups, with a value of *p* < 0.05 deemed significant. All data are expressed as the mean ± SEM (Standard Error of the Mean).

## Results

### Results of network pharmacology experiments

#### Target prediction of naringin for the treatment of post-infectious cough

**Fig. 1:**
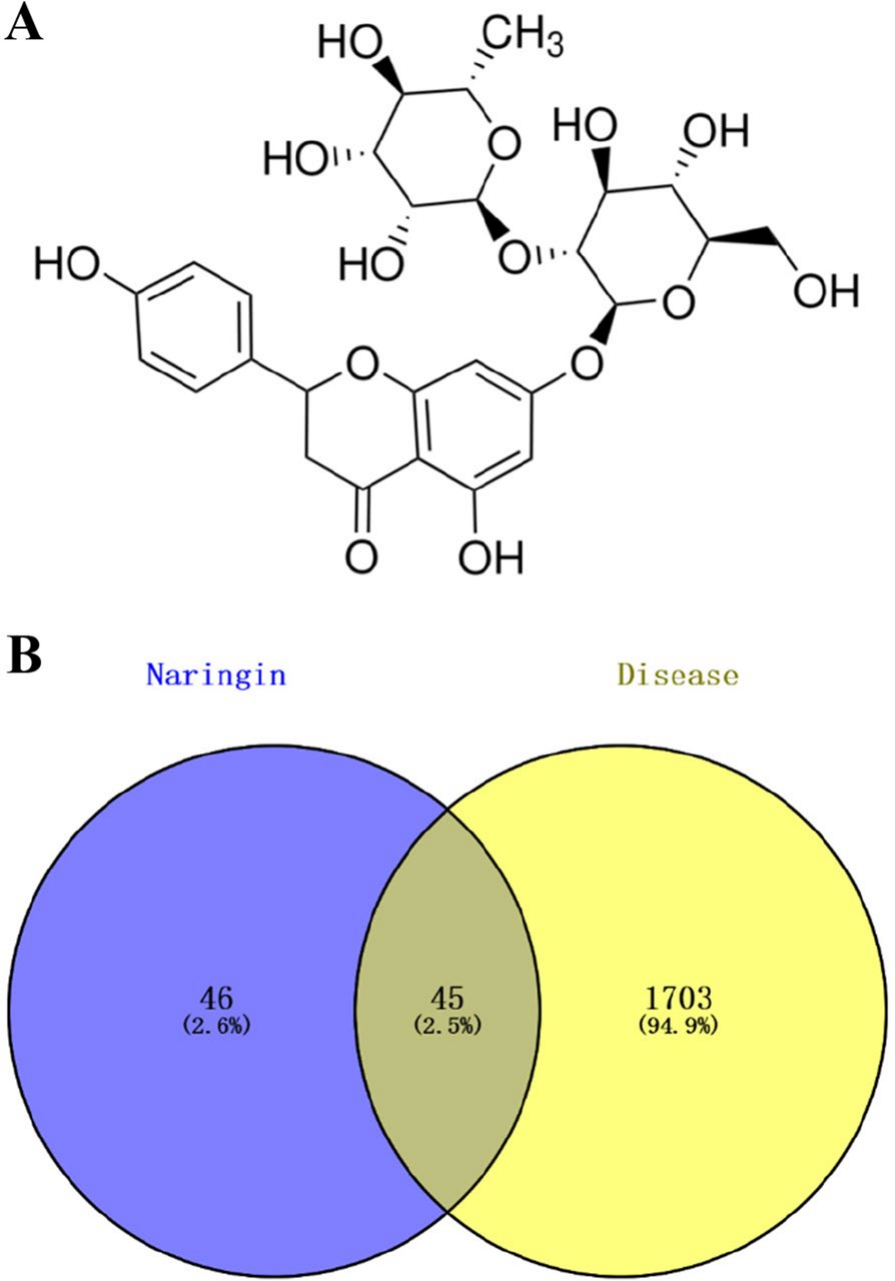
**A** Chemical structure of naringin (PubChem CID: 442,428). **B** Venn diagram of active ingredient targets and disease targets of naringin. The middle part is the 45 intersecting targets

The chemical structure of naringin (Fig. 1B) was searched in the PubChem database, and related target information was obtained from the Swiss Target Prediction and Super-PRED databases. Targets related to post-infectious cough were identified through the GeneCards and OMIM databases. After removing duplicates, the PIC-related targets were mapped to the corresponding targets of naringin, resulting in 45 common genes, as shown in Fig. 1B. These genes represent the predicted targets for naringin in the treatment of PIC. The number of targets retrieved from each database is summarized in Table 2.

**Table 2:** Database sources and target counts for post-infectious cough and naringin

Database	Number of Targets
GeneCards	1725
OMIM	23
SuperPred	90
SwissTargetPrediction	1

#### Visualizing the disease-component-target network

**Fig. 2:**
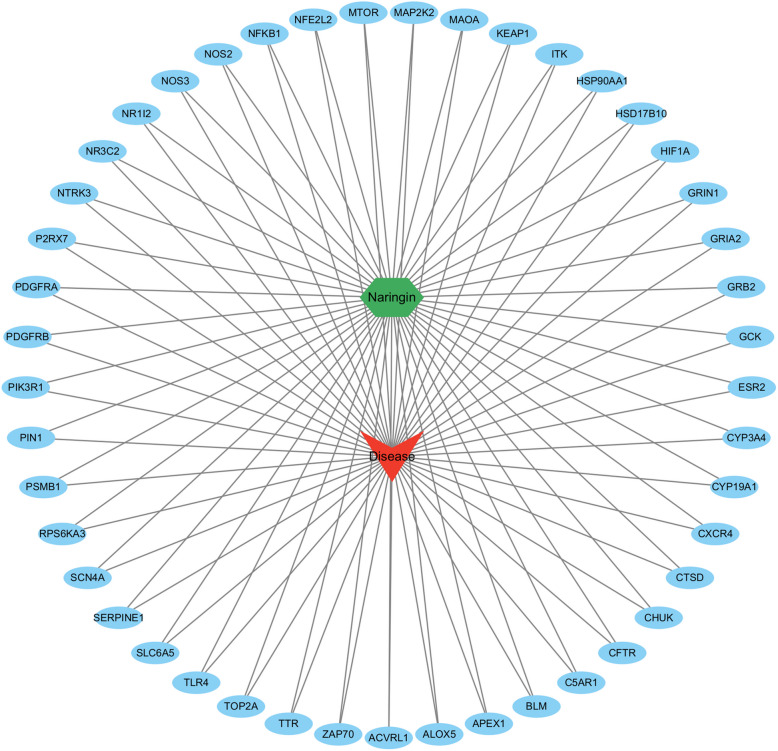
“Disease-Component-Target” interaction network. Red arrows represent diseases, blue circles represent intersecting gene targets, and green hexagons represent naringin

Cytoscape software was used to create an interaction network of "Disease-Component-Target". As shown in Fig. 2, the blue oval represents the intersecting gene targets of naringin and the disease. The network contains 47 nodes and 90 interaction edges.

#### Protein–protein interaction analysis

**Fig. 3:**
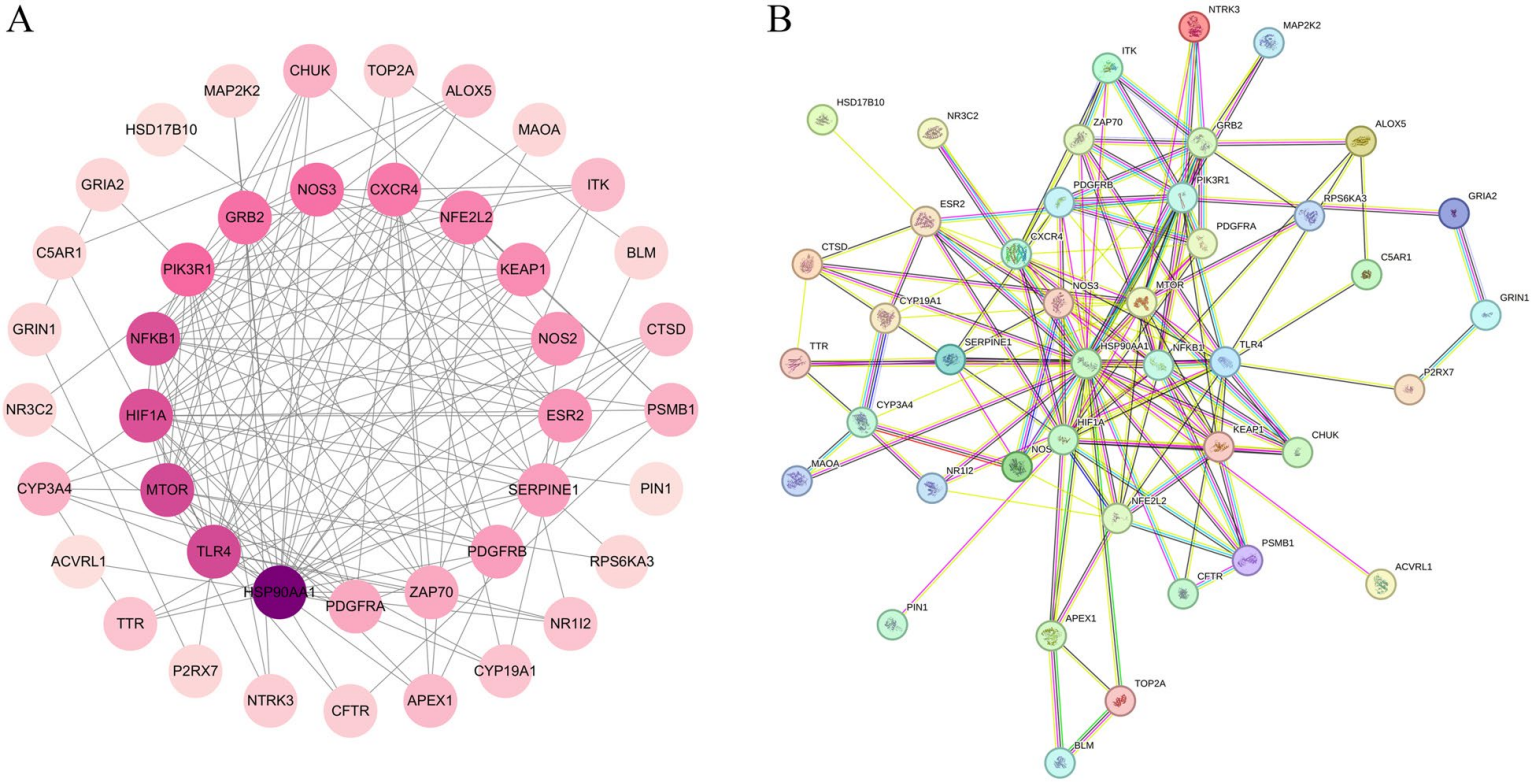
PPI network diagram. **A** The circle indicates the intersection target, the darker the color of the circle, the larger the Degree value of the target. **B** The circles represent different proteins, and the connecting lines between the circles indicate the interactions between the proteins

The 45 intersecting target proteins of naringin and post-infectious cough were imported into the STRING database, with the interaction score set to 0.4. Discrete points were hidden, and the resulting PPI network was imported into Cytoscape for visualization. As shown in Fig. 3, the color intensity corresponds to the degree value, with darker colors indicating higher degree values. The network contains 42 nodes and 149 edges, illustrating the relationship and importance ranking of the core targets in the protein–protein interaction network.

#### GO gene enrichment and KEGG pathway analysis

**Fig. 4:**
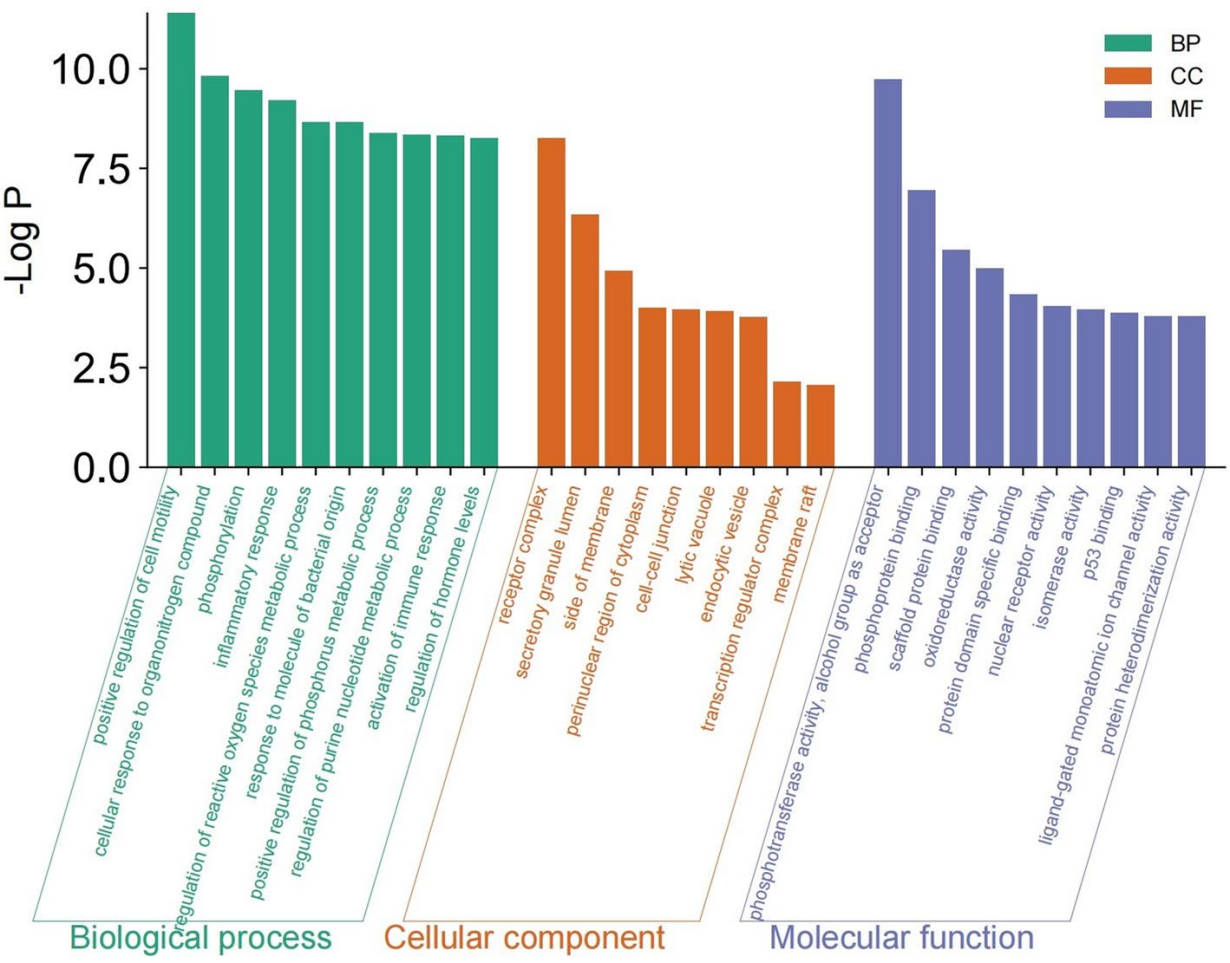
GO functional enrichment analysis. The X-axis showed enriched gene ontology categories of the targets, and the Y-axis showed the enrichment scores of these terms

GO functional analysis of the 45 intersecting targets, including Biological Processes, Cellular Components, and Molecular Functions, was performed using the Metascape database. As shown in Fig. 4, the top ten results in terms of Log *P*-value are presented in the bar chart below, with all results less than 10 listed. The GO-BP results mainly include the positive regulation of cell motility, cellular response to organonitrogen compounds, and others. The GO-CC results mainly include receptor complexes, secretory granule lumens, and others. The GO-MF results primarily involve phosphotransferase activity, alcohol group as acceptor, phosphoprotein binding, and others.

**Fig. 5:**
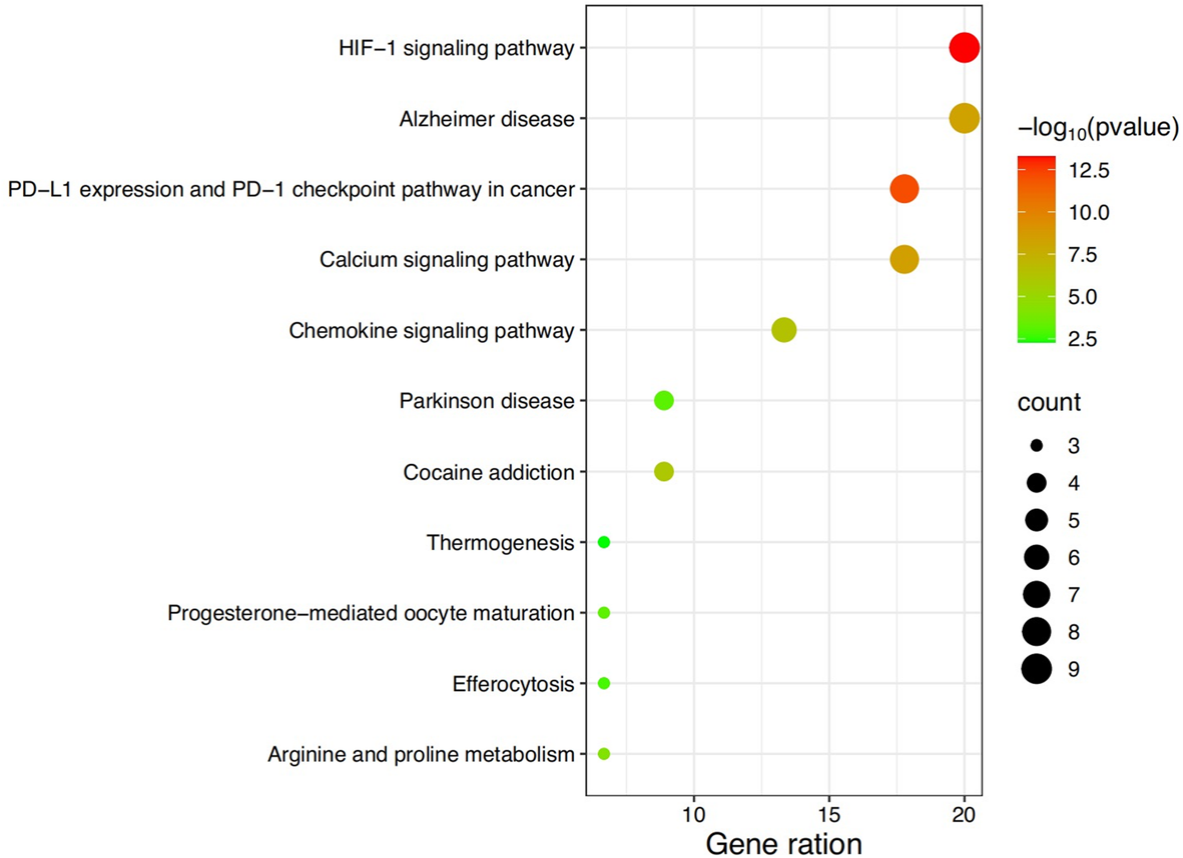
KEGG pathway enrichment analysis. The X-axis represents the gene ratio (number of overlapping targets in a pathway divided by total genes in that pathway), the Y-axis displays enriched pathways. Dot size corresponds to the count of overlapping targets, and color intensity reflects the -log10 (*p*-value) significance level. Top 10 enriched pathways are shown

The 45 intersecting targets were then analyzed for pathway enrichment using the Metascape database. As shown in Fig. 5, a total of 11 signaling pathways were enriched. The main pathways included the HIF-1 signaling pathway, Alzheimer’s disease, PD-L1 expression and PD-1 checkpoint pathway in cancer, and others.

#### Docking results of naringin with key target proteins

**Fig. 6:**
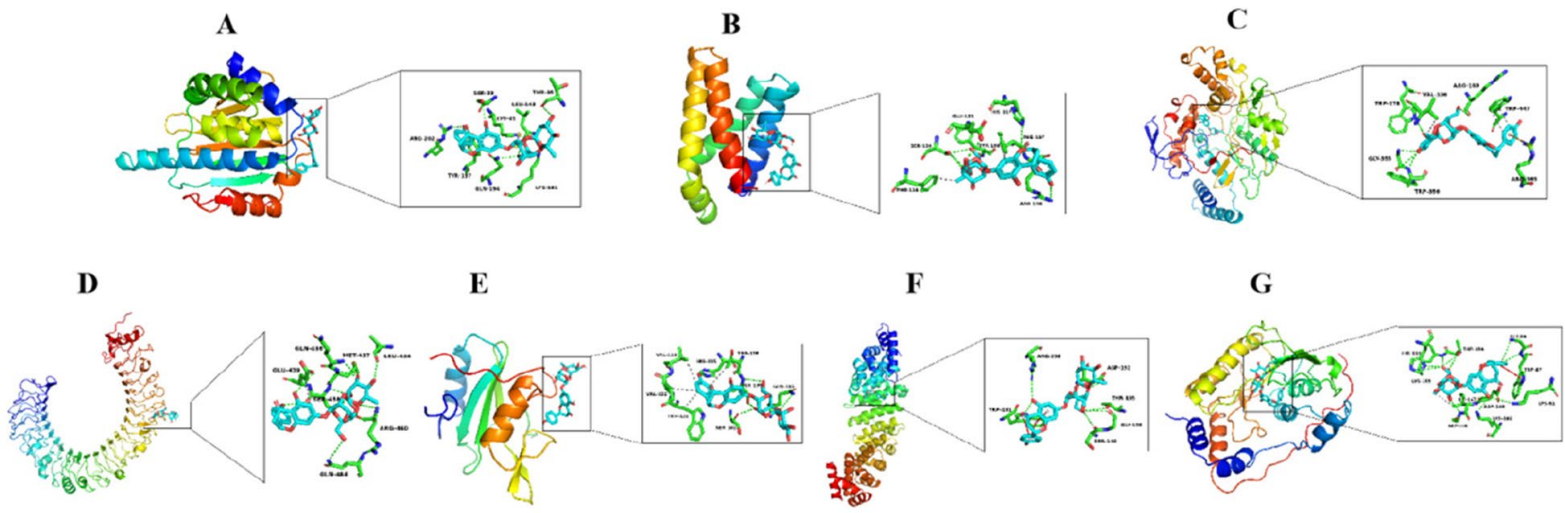
Molecular docking of naringin to key target proteins. **A** Naringin with HSP90AA1. **B** Naringin with MTOR. **C** Naringin with NOS3. **D** Naringin with TLR4. **E** Naringin with HIF1A. **F** Naringin with NF-κB. **G** Naringin with GRB2

The key targets HSP90AA1, TLR4, MTOR, HIF1A, NF-κB1, NOS3, and GRB2 were selected for molecular docking with naringin. The Degree, Closeness, and Betweenness centrality values of these key targets are detailed in Table 3. The binding energies for these interactions are summarized in Table 4, with all docking energies below −6 kcal/mol, indicating a strong affinity between naringin and the screened core targets. The target-drug combinations with the lowest binding energies were visualized using PyMOL software and are presented in Fig. 6.

**Table 3:** Network centrality metrics of seven key targets

Target	Degree	Betweenness	Closeness
HSP90AA1	28	581.06	34.33
TLR4	18	255.57	29.17
MTOR	18	169.93	29
HIF1A	17	146.04	28.67
NF-κB1	17	70.75	28.67
NOS3	14	153.97	27.17
GRB2	13	79.81	26.17

**Table 4:** Binding energies of naringin docked to seven key targets

Docking target	PDB	Binding energy (kcal/mol)	Grid Dimensions (Å)	Grid Center (x, y, z)
HSP90AA1	4BQG	−7.7	47.25 × 47.25 × 47.25	0.343, 14.836, 20.605
TLR4	4G8A	−7.4	85.76 × 87.15 × 87.15	−18.684, −1.014, −57.735
MTOR	3FAP	−7.8	47.25 × 47.0 × 47.25	−18.102, 29.181, 25.388
HIF1A	8HE3	−6.9	47.25 × 47.25 × 47.25	30.737, 21.094, 9.982
NF-κB1	5ULP	−8.4	75.25 × 75.25 × 75.25	−1.478, −21.243, 0.416
NOS3	3NOS	−10.2	55.65 × 55.65 × 55.65	23.646, 9.279, 22.928
GRB2	1BMB	−9.8	47.25 × 47.25 × 47.25	5.269, 1.383, 27.523

### Results of animal experiments

#### General condition of guinea pigs

The guinea pigs in the control group exhibited good overall health, characterized by a normal mental state, smooth and shiny fur, regular food intake, and even respiration. Following the modeling process, the guinea pigs in all groups displayed signs of poor mental condition, including disheveled fur, reduced fur gloss, diminished food intake, and increased respiratory rate. After exposure to citric acid aerosol stimulation, the guinea pigs developed noticeable symptoms such as coughing, face scratching, sneezing, and rapid breathing. However, after drug treatment, their symptoms showed significant improvement.

#### Effect of naringin on cough sensitivity in guinea pigs on the seventh day after viral infection

**Fig. 7:**
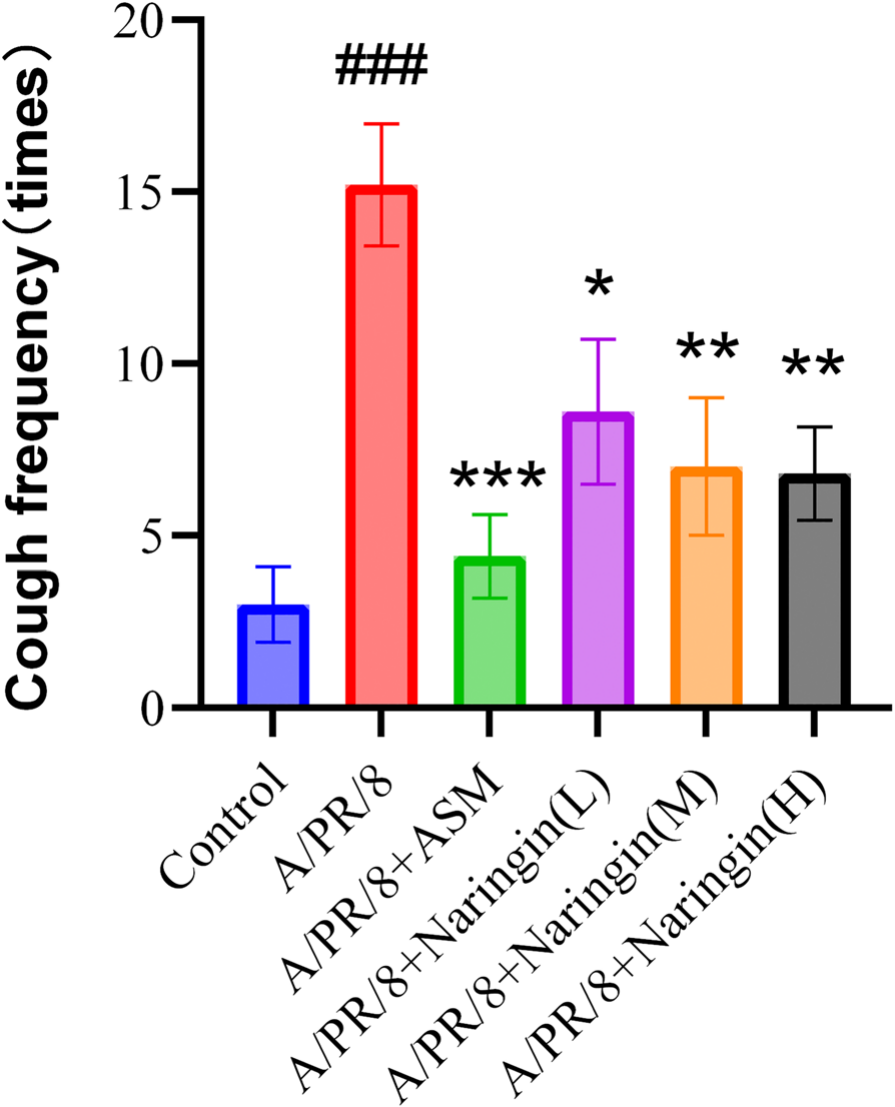
The cough frequency in each group on the seventh day after viral infection (*n* = 5–6). Data were shown as mean ± SEM. ### *p* < 0.001, compared with the control group. * *p* < 0.05, ** *p* < 0.01, compared with A/PR/8 group

As shown in Fig. 7, the number of coughs on the seventh day post-infection was significantly higher in the model group compared to the control group. In contrast, the naringin group exhibited a significant reduction in the number of coughs, particularly in the medium and high-dose subgroups.

#### Effect of naringin on the relative expression of inflammatory factors in guinea pig lung tissue on the seventh day after infection

**Fig. 8:**
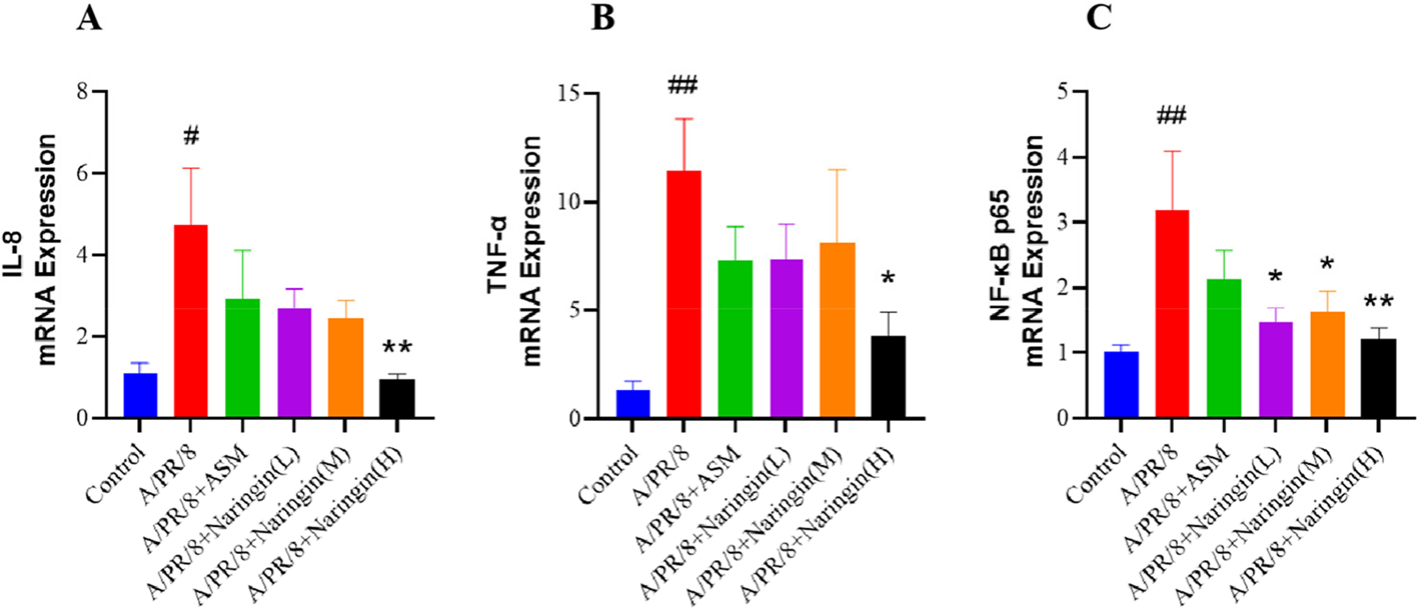
**A**-**C** The mRNA levels of IL-8, TNF-α and NF-κB p65 in the lung tissues were determined by RT-qPCR (*n* = 6). Data were shown as mean ± SEM. # *p* < 0.05, ## *p* < 0.01, ### *p* < 0.001, compared with the control group. * *p* < 0.05, ** *p* < 0.01, compared with A/PR/8 group

As shown in Fig. 8, the relative expression of IL-8, TNF-α, and NF-κB p65 mRNA was significantly higher in the model group compared to the control group. However, the relative expression of these markers was significantly reduced in the naringin group compared to the model group.

#### Pathologic effects of naringin on the lung tissue of guinea pigs on the seventh day after infection

**Fig. 9:**
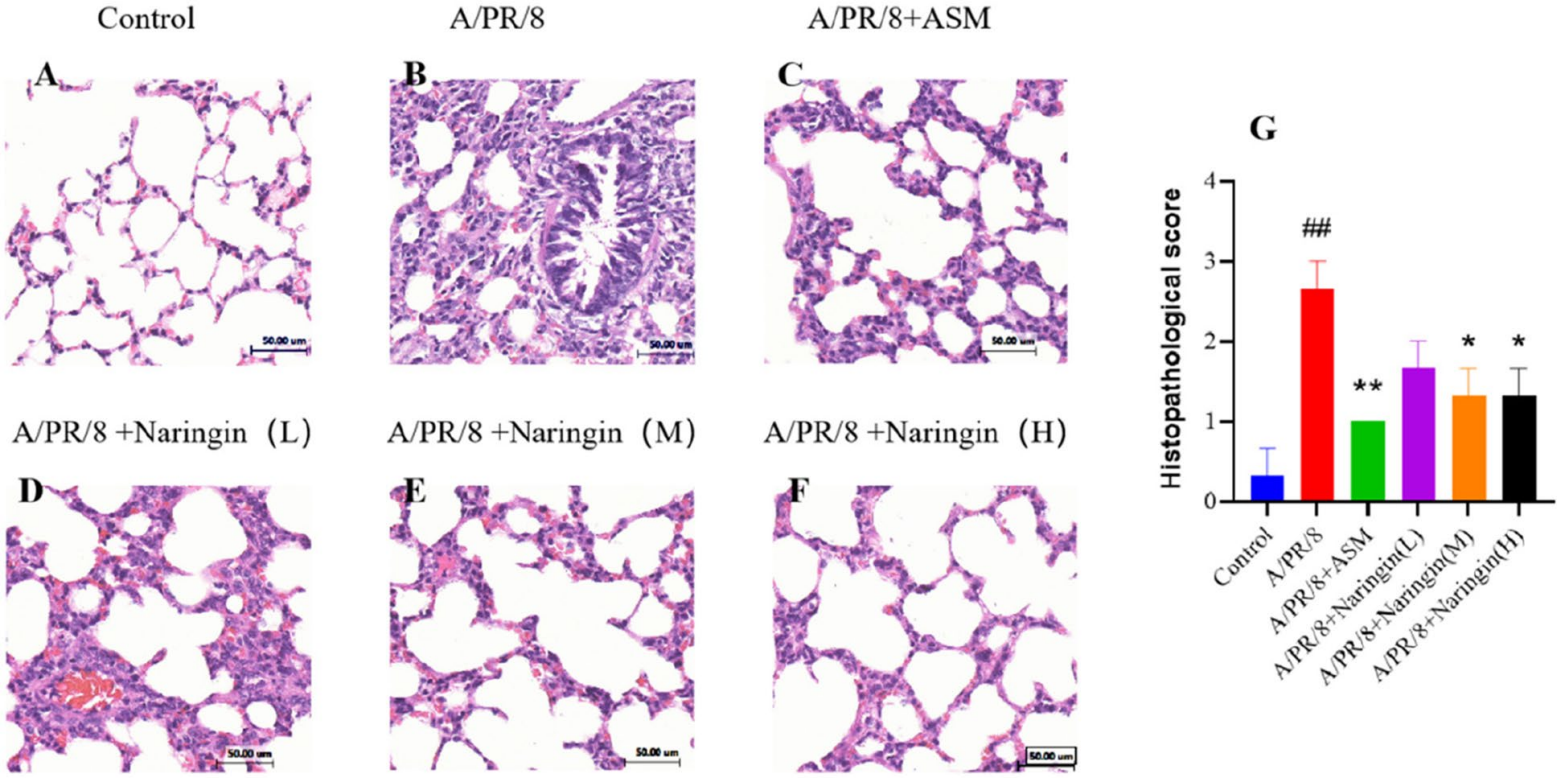
**A**-**F** Histological observations of lung tissues for guinea pigs (*n* = 3). (Scale bar = 50 μm). **G** Histopathologic scoring of guinea pig lung tissue. Data were shown as mean ± SEM. ## *p* < 0.01, compared with the control group. * *p* < 0.05, ** *p* < 0.01, compared with A/PR/8 group

As shown in Fig. 9, the alveolar structure in the normal group remained intact without inflammatory cell infiltration. In contrast, the model group displayed significant pathological changes, including extensive inflammatory cell infiltration. Treatment with naringin significantly improved lung pathology, with reduced inflammatory cell infiltration and largely preserved alveolar structure.

#### Effect of naringin on SOD activity in guinea pig lung tissue on the seventh day after infection

**Fig. 10:**
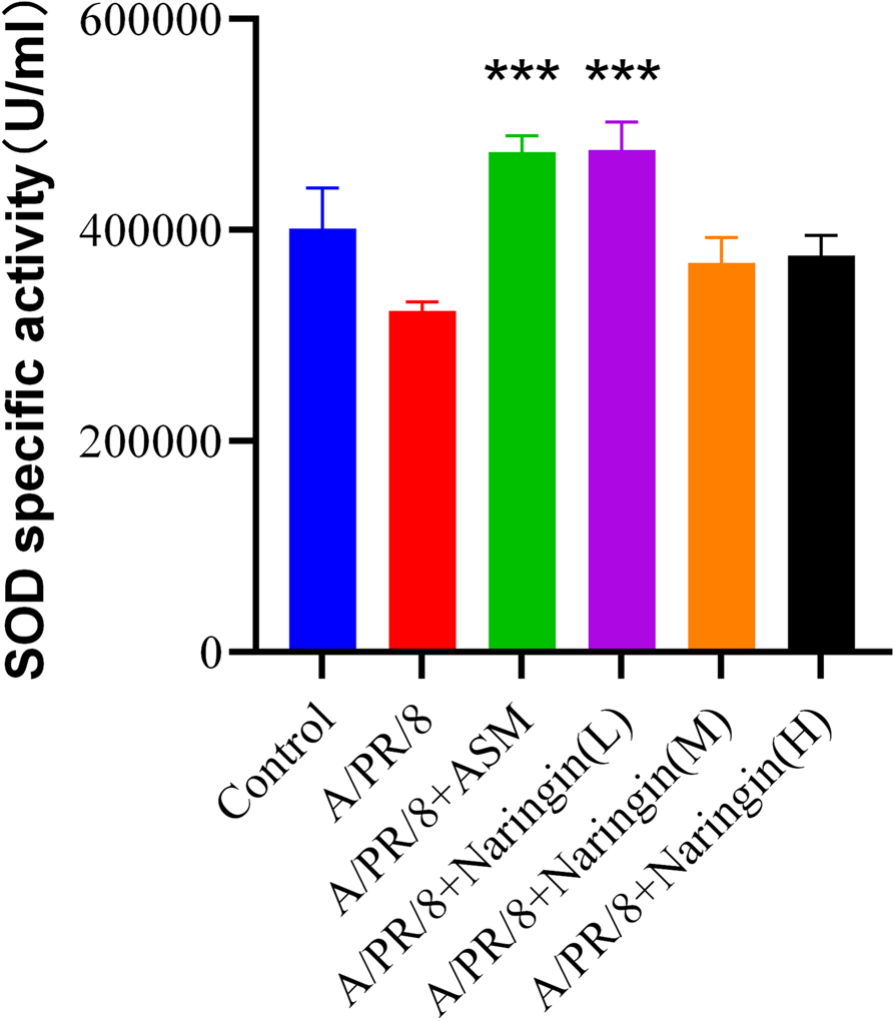
The activity of SOD in lung tissue was measured by colorimetric assay (*n* = 5–6). Data were shown as mean ± SEM. *** *p* < 0.001, compared with A/PR/8 group

As show in Fig. 10, compared with the control group, lung tissue SOD activity was decreased in the virus group, although this difference was not statistically significant. However, the naringin group showed a significant increase in lung tissue SOD activity compared to the virus group.

## Discussion

Currently, Western medicine lacks targeted therapeutic strategies for PIC and primarily relies on symptomatic treatment. Commonly used medications, including antihistamines, central antitussive agents, and anti-inflammatory drugs, can alleviate symptoms but often exhibit limited efficacy [1]. The development of Chinese patent medicines for PIC also remains inadequate. Among the few available options, Suhuang Zhike Capsule is primarily indicated for PIC with the wind-cold syndrome, which fails to address the needs of patients with other syndrome types.

Medical research indicates that the pathogenesis of PIC mainly involves widespread airway inflammation, epithelial cell damage, airway hyperresponsiveness, and neurogenic inflammation. Inflammatory mediators, such as TNF-α, IL-6 (Interleukin-6), IL-8, and IL-10 (Interleukin-10) [16–18], along with neurotransmitters, play critical roles in these processes. The flavonoid naringin, utilized in this study, has been widely reported for its anti-inflammatory properties. For instance, research by Kim et al. demonstrated that naringin exerts significant inhibitory effects on pulmonary inflammation induced by acrolein inhalation in mice by modulating MAPK (Mitogen-Activated Protein Kinase), p53 (Tumor Protein p53), and NF-κB signaling pathways, thereby reducing IL-1β and TNF-α expression [19]. Similarly, Xiong et al. observed that naringin suppressed inflammatory cell infiltration in the lungs and reduced IL-4 (Interleukin-4) levels in BALF (Bronchoalveolar Lavage Fluid) in an ovalbumin-induced asthma mouse model [20]. Furthermore, Ahmad et al. found that naringin inhibited the expression of iNOS (Inducible Nitric Oxide Synthase), COX-2 (Cyclooxygenase-2), ICAM-1 (Intercellular Adhesion Molecule-1), MIP-2 (Macrophage Inflammatory Protein-2), and PGE2 (Prostaglandin E2) mRNA in a carrageenan-induced pleurisy model. It also suppressed COX-2 protein expression and NF-κB p65 activation [21]. Research from Su Weiwei's team at Sun Yat-sen University revealed that naringin significantly alleviated pulmonary edema, structural damage, and neutrophil infiltration in acute lung injury models induced by LPS (Lipopolysaccharide). It also inhibited MPO (Myeloperoxidase) activity, iNOS activity, and the production of TNF-α and IL-8 in lung tissues [22, 23]. Collectively, these studies suggest that naringin achieves its therapeutic effects on PIC by suppressing inflammatory mediators.

Through PPI analysis, key targets of naringin for PIC treatment were identified, including HSP90AA1, TLR4, MTOR, HIF1A, and NF-κB1. HSP90AA1, a heat shock protein, is essential for maintaining protein folding, cellular homeostasis, and stress responses. It stabilizes inflammatory signaling molecules such as NF-κB, thereby promoting the release of inflammatory mediators [24]. TLR4, a pattern recognition receptor, recognizes PAMPs (Pathogen-Associated Molecular Patterns) like lipopolysaccharides and activates innate immune responses [25]. In PIC, excessive TLR4 activation can trigger airway inflammation, leading to a significant release of cytokines such as IL-8 and TNF-α [26]. Inhibiting TLR4 signaling effectively reduces inflammatory cytokine levels and alleviates airway inflammation [27]. MTOR is central to regulating cell growth, metabolism, and inflammation, enhancing antioxidative capacity and mitigating oxidative damage in airway tissues [28]. NF-κB1, a key transcription factor in the classical inflammatory signaling pathway, is closely associated with the expression of cytokines like IL-1β and TNF-α [29] Persistent NF-κB1 activation contributes to chronic inflammation and tissue damage in PIC [30, 31]. Suppression of NF-κB1 signaling can significantly reduce inflammatory mediator release, thereby mitigating airway inflammation.

In addition, KEGG enrichment pathway analysis revealed that the HIF-1 signaling pathway may be a key pathway for naringin in treating post-infection cough. This pathway encompasses key targets HIF1A and NF-κB1. It was demonstrated that viral infections can activate hypoxia-inducible factor HIF-1α (encoded by HIF1A) by inducing local tissue hypoxia [32]. HIF-1α forms a synergistic regulatory network with TLR4/NF-κB1: TLR4 enhances the stability of HIF-1α through the MyD88-dependent signaling pathway, forming a"HIF-1α → TLR4 → HIF-1α"positive feedback loop. Simultaneously, HIF-1α activates the NF-κB pathway by inhibiting prolyl hydroxylase, driving the release of pro-inflammatory factors such as TNF-α and IL-8 and aggravating the inflammatory response [33]. Accordingly, this study measured the relative mRNA expression levels of IL-8, IL-1β, TNF-α, and NF-κB1, along with SOD activity in lung tissues from different groups of guinea pigs to elucidate the mechanisms by which naringin treats PIC.

The pharmacological effects of naringin were further confirmed in animal experiments. Results demonstrated that naringin significantly reduced cough frequency in guinea pigs, improved pathological changes in lung tissues, and exhibited potent antitussive effects. From the perspective of inflammatory mediators, naringin markedly decreased the mRNA expression of IL-8, IL-1β, TNF-α, and NF-κB1, indicating its strong anti-inflammatory properties. Moreover, naringin significantly enhanced SOD activity, suggesting its antioxidative potential to mitigate oxidative stress-induced lung damage. These findings suggest that the dual mechanisms of anti-inflammatory and antioxidative effects may be critical to naringin's therapeutic efficacy in treating PIC.

Although this study provides novel insights into the therapeutic potential of naringin for PIC, several limitations should be acknowledged. First, the findings are based on a guinea pig model, which may not fully recapitulate the complex pathophysiology of human post-infectious cough. Species-specific differences in immune responses or drug metabolism could influence the translational implications of these results. Second, the small sample size (*n* = 5–6 per group) reduced statistical power, particularly in interpreting borderline outcomes such as the non-significant reduction in SOD activity between the model and control groups. Future studies should incorporate larger cohorts to strengthen translational relevance and utilize human-derived cells or tissues to validate preclinical findings and refine therapeutic strategies.

In conclusion, this study demonstrated that naringin has therapeutic potential to alleviate PIC symptoms in a guinea pig model through anti-inflammatory and antioxidative mechanisms. These preclinical findings provide support for naringin as a potential candidate derived from Chinese medicine for PIC treatment. Future work will combine clinical studies and modern drug development technologies to further validate and promote the application of this natural compound in respiratory disease therapy.

## Data Availability

The raw experimental datasets generated during this study will be deposited in the Figshare repository (https://figshare.com/) upon manuscript acceptance. Processed data supporting the findings are included in this article.
